# Plasma trimethylamine N-oxide and arteriosclerotic cardiovascular disease: a systematic review and meta-analysis of case–control studies

**DOI:** 10.3389/fcvm.2026.1913190

**Published:** 2026-09-25

**Authors:** Yang Ou, Jiuhua She, Boyan Gao, Jiayong Liu, Yang Yu, Wenxin Chen, Yifan Wang, Meiqi Ren, Guijun Shi

**Affiliations:** 1 College of Chinese Medicine, Changchun University of Chinese Medicine, Changchun, China; 2 General Internal Medicine, The Affiliated Hospital of Changchun University of Chinese Medicine, Changchun, Jilin, China; 3 Department of Cardiology, Changchun Chinese Medicine Hospital, Changchun, Jilin, China; 4 Changchun University of Chinese Medicine, Changchun, Jilin, China

**Keywords:** atherosclerotic cardiovascular disease, case–control study, gut microbiota, meta-analysis, trimethylamine N-oxide

## Abstract

**Introduction:**

Trimethylamine N-oxide (TMAO), a gut microbiota-derived metabolite, has been implicated in atherosclerosis. However, the association between plasma TMAO levels and atherosclerotic cardiovascular disease (ASCVD) risk remains inconsistent across studies.

**Methods:**

We searched PubMed, Embase, Cochrane, and Web of Science for case-control (including nested) studies reporting odds ratios (ORs) with 95% confidence intervals (CIs) for TMAO and ASCVD. Estimates were pooled under fixed- or random-effects assumptions according to the observed heterogeneity, supplemented by subgroup, sensitivity, and publication bias assessments.

**Results:**

Nine studies were included in this meta-analysis. Elevated TMAO levels (highest vs. lowest quantile) were significantly associated with ASCVD risk (OR = 1.39; 95% CI: 1.21–1.61; *I*^2^ = 28.8%). The continuous analysis showed a significant pooled standardized mean difference (SMD) of 0.43 (95% CI: 0.14–0.71), but with extreme heterogeneity (*I*^2^ = 93.4%). The association was stronger in the primary prevention (OR = 1.51; 95% CI: 1.28–1.78) than in the secondary prevention cohorts (OR = 1.10; 95% CI: 0.83–1.46; *P* for interaction = 0.057). GRADE (Grading of Recommendations Assessment, Development, and Evaluation) certainty was low for the categorical analysis and very low for the continuous analysis.

**Discussion:**

This meta-analysis showed that high versus low plasma TMAO levels were significantly associated with increased ASCVD risk (OR = 1.39; 95% CI: 1.21–1.61), particularly in primary prevention populations (OR = 1.51; 95% CI: 1.28–1.78), and supported TMAO as a potential risk indicator for primary prevention. However, continuous analysis yielded highly unstable estimates with extreme heterogeneity (*I*^2^ = 93.4%), precluding linear dose-response conclusions, and the certainty of evidence remained low to very low. Current evidence does not yet endorse routine clinical screening but lends credence to gut microbiota-targeted preventive strategies, such as dietary modifications aimed at reducing the intake of TMAO precursors from red and processed meat. Future standardized prospective studies are necessary to establish the clinical utility of TMAO.

**Systematic Review Registration:**

PROSPERO CRD420261357905.

## 1 Introduction

Cardiovascular disease (CVD) is the leading cause of death and disease burden worldwide, with atherosclerotic cardiovascular disease (ASCVD) accounting for the majority of cases. Despite significant progress in controlling traditional risk factors, the residual risk of ASCVD persists, driving researchers to continuously explore novel pathogenic pathways and biomarkers (1). In recent years, the relationship between gut microbiota metabolites and cardiovascular disease has become a research hotspot, with trimethylamine N-oxide (TMAO) attracting particular attention. TMAO is primarily derived from dietary choline, L-carnitine, and betaine nutrients, which are abundantly present in red meat, processed meat, eggs, and dairy products (2). These are converted by the gut microbiota into trimethylamine (TMA), which is subsequently oxidized to TMAO in the liver by flavin-containing monooxygenase 3 (FMO3) (3). In this landmark study, Tang et al. first demonstrated that elevated plasma TMAO levels were associated with more than a twofold increased risk of major adverse cardiovascular events (MACE) in a stable cohort of patients undergoing elective coronary angiography, establishing TMAO as an independent predictor of cardiovascular risk. Notably, L-carnitine is found in concentrations approximately four times higher in red meat than in poultry or fish (4), and habitual red meat ingestion increases plasma TMAO levels more than other animal or plant-based protein sources (3). This dietary precursor–microbiota–host axis positions the consumption of red and processed meats as a potentially modifiable risk factor for cardiovascular disease through TMAO elevation. Numerous studies have demonstrated that TMAO promotes atherosclerosis progression through multiple mechanisms, including endothelial dysfunction, activation of the NLRP3 inflammasome, foam cell formation, platelet hyperreactivity, and vascular calcification (1, 5, 6).

Multiple meta-analyses have confirmed the association between TMAO and the risk of cardiovascular events. A meta-analysis of 30 prospective studies demonstrated that high TMAO levels are significantly associated with the risk of MACE [hazard ratio (HR) = 1.41, 95% confidence interval (CI): 1.20–1.54] (7). Another meta-analysis focusing on patients with myocardial infarction similarly found that elevated TMAO levels were significantly associated with the risk of MACE (risk ratio (RR) = 1.94, 95% CI: 1.39–2.73) and all-cause mortality (RR = 1.56, 95% CI: 1.00–2.44) (8). However, a recent umbrella review systematically evaluating 27 meta-analyses found that although most studies supported an association between TMAO and cardiovascular disease, the original studies predominantly enrolled high-risk populations and lacked prospective studies in healthy populations. Moreover, existing systematic reviews have serious methodological quality deficiencies, resulting in generally low credibility of the evidence (5, 9). Furthermore, a recent prospective cohort study in a community-based population, the Multi-Ethnic Study of Atherosclerosis (MESA), showed that TMAO was dose-dependently associated with ASCVD events, but the effect size was more modest than that in previous studies in high-risk populations (HR = 1.33, 95% CI: 1.02–1.74 for highest vs. lowest quintile) (9), suggesting that population characteristics may significantly influence effect estimates.

Notably, TMAO can be measured in various biological media, including plasma, serum, urine, and feces. The metabolic implications reflected by TMAO levels and the strength of their association with disease may differ across different media (6). Existing meta-analyses have often not strictly distinguished between measurement media when including studies or have primarily focused on serum samples. However, plasma TMAO, one of the most common detection media in clinical research, has not yet been systematically integrated into case–control study designs regarding its stability and strength of association with ASCVD (5). Furthermore, previous meta-analyses have mostly included cohort study designs. However, case–control studies (including traditional and nested case–control studies), an important type of observational study design, have a unique value, particularly in exploring rare outcomes or when retrospective exposure assessment is needed, and can synthesize evidence more efficiently across different populations, TMAO measurement methods, and disease subtypes (1, 7). However, there is currently a lack of meta-analyses specifically targeting case–control study designs to systematically evaluate the association between plasma TMAO levels and ASCVD.

Therefore, this study aimed to systematically retrieve existing case–control studies, integrate the evidence on the association between plasma TMAO levels and ASCVD risk through meta-analysis, and explore the impact of factors such as study design, population characteristics, and effect size type on the results to provide a more comprehensive evidence-based basis for the clinical value of TMAO as a biomarker for ASCVD risk.

## 2 Study

### 2.1 Methods

#### 2.1.1 Protocol and registration

The protocol for this study was registered with the International Prospective Register of Systematic Reviews (PROSPERO; registration number: CRD420261357905). This systematic review and meta-analysis were conducted in accordance with the Preferred Reporting Items for Systematic Reviews and Meta-Analyses (PRISMA) statement (10). All inclusion criteria, prespecified primary outcomes, planned subgroup classifications, and overall analysis strategies were consistent with the registered protocol.

#### 2.1.2 Eligibility criteria

The inclusion criteria of this study followed the PECOS principle (participants, exposure, comparator, outcomes, and study design).

##### 2.1.2.1 Inclusion criteria

**Population**

Cases: Patients with clinically or imaging-confirmed ASCVD, including but not limited to coronary heart disease (CHD), myocardial infarction, acute coronary syndrome, ischemic stroke, and carotid atherosclerosis.

Controls: Individuals without a definite history of ASCVD, who could be healthy populations or those confirmed to be free of atherosclerosis by coronary angiography or ultrasonography.

**Exposure and comparison**

Exposure: Plasma TMAO concentration.

Comparison: Comparison between different TMAO level groups (e.g., highest quantile vs. lowest quantile or per 1-unit or 1-SD increase). Studies had to report odds ratios (ORs) with 95% CIs or provide raw data from which ORs could be calculated.

**Outcomes**

Primary outcome: ASCVD events [including myocardial infarction, stroke, coronary artery disease (CAD), acute coronary syndrome, and unstable angina pectoris (UAP)].

Secondary outcomes: Atherosclerosis-related complications (e.g., cardiac death and heart failure) or recurrent ASCVD events.

**Study design**

Case–control studies (including traditional case–control studies, nested case–control studies, and composite study designs containing case–control studies) were included.

##### 2.1.2.2 Exclusion criteria

**Ineligible population**

Studies were excluded if the controls had a definite history of ASCVD or did not clearly exclude ASCVD, or if the cases were not definitely diagnosed with ASCVD or had unclear diagnostic criteria.

**Ineligible exposure or outcome**

Studies were excluded if plasma TMAO concentration was not measured; if the OR for the TMAO-ASCVD association was not reported; or if the exposure was non-plasma TMAO (e.g., urine or fecal TMAO) or TMAO precursors (e.g., choline and carnitine) reported alone.

**Ineligible study design**

Reviews, meta-analyses, animal studies, cell studies; cross-sectional studies; pure cohort studies (without case–control analysis); case reports, case series, and conference abstracts were excluded.

**Incomplete data**

Studies were excluded if valid data could not be extracted (e.g., only *P*-values reported without providing raw data to calculate OR and 95% CI). Duplicate publications were excluded, and only the most recent or complete versions were included.

#### 2.1.3 Search strategy

We systematically searched four databases (PubMed, Embase, Cochrane, and Web of Science) using the following Medical Subject Headings (MeSH) and accessible search terms: (“TMAO” OR “trimethylamine oxide” OR “trimethylammonium oxide” OR “trimethylamine N-oxide”) AND (“atherosclerotic cardiovascular disease” OR “ASCVD” OR “cardiovascular events” OR “MACE” OR “cardio vascular” OR “coronary artery disease” OR “CAD” OR “CHD” OR “stroke”) AND (“Case-Control Studies”[MeSH] OR “case-control” OR “case control” OR “cases-controls” OR “case-control study” OR “case control study”). No publication year restrictions were imposed. During the study selection process, two independent reviewers rigorously evaluated the articles (titles, abstracts, and full texts). Disagreements were resolved through discussion or consultation with a third reviewer.

#### 2.1.4 Study selection

Two investigators independently extracted relevant study information using a customized data collection form in Excel. A third reviewer verified the extracted baseline characteristics (first author, publication year, country, study design, disease type, sample size, sex, etc.). Two independent reviewers assessed the methodological quality of the studies. The Newcastle–Ottawa Quality Assessment Scale, which includes three quality parameters (selection, comparability, and outcome, comprising eight specific items), was used to evaluate study quality. A third expert resolved NY disagreements.

#### 2.1.5 Statistical analysis

Statistical analyses were performed using STATA 12.0 software.

Analysis strategy: Owing to the inherent differences in effect measure types, effect estimates from categorical comparisons (e.g., highest vs. lowest quantile) and continuous associations (e.g., per 1-SD or 1-unit increase) were analyzed separately using independent models.

Effect measures: For categorical variables, the OR with 95% CI comparing the highest and lowest quantiles was used. For continuous variables, the OR with a 95% CI per 1-unit increase was used as the primary measure. When unit conversion was not feasible, the standardized mean difference (SMD) was employed as an alternative effect measure. All data were obtained from the most fully adjusted models. Heterogeneity was assessed using the Cochrane Q test and *I*^2^ statistic. When *P* ≥ 0.1 and *I*^2^ < 50%, no significant statistical heterogeneity was indicated, and a fixed-effect model was used. When *P* < 0.1 and *I*^2^ ≥ 50%, significant heterogeneity was indicated, and a random-effects model was used for the pooled analysis.

Subgroup analyses were performed to explore the potential sources of heterogeneity. Prespecified subgroup analyses were conducted when at least two studies were available. The prespecified subgroup factors included disease type, region, race, population type, study design, age, measurement unit, and measurement method.

A sensitivity analysis was performed using the leave-one-out method. Publication bias was assessed using funnel plots and Egger's test. A *P*-value < 0.05 was considered statistically significant.

#### 2.1.6 Certainty of evidence assessment

The certainty of the evidence for each outcome was assessed using the Grading of Recommendations Assessment, Development, and Evaluation (GRADE) framework (11). According to GRADE, evidence from observational studies begins with low certainty. Certainty ratings were then evaluated across five domains: risk of bias, inconsistency, indirectness, imprecision, and publication bias. For the categorical analysis, the evidence could be upgraded to a moderate-to-large effect size if justified. A Summary of Findings (SoF) table was constructed using the GRADEpro GDT software (McMaster University, Hamilton, ON, Canada) to present the relative and absolute effect estimates alongside the certainty ratings for the primary outcomes.

### 2.2 Results

#### 2.2.1 Study selection

A total of 154 studies were identified through database searches (PubMed = 48, Web of Science = 29, Cochrane = 0, Embase = 77). The last search was performed on 15 January 2026. After removing 48 duplicate records, 106 studies remained for the initial screening. Following the screening of titles and abstracts, 74 studies were excluded, and the full texts of the remaining 32 articles were assessed for eligibility. Among these, four studies were excluded because the full text could not be obtained, five were non-case–control studies, five had ineligible study designs (including inconsistent exposure or outcomes or controls with prior ASCVD), four were duplicate publications (including preliminary experiments and conference reports of the same study), and five had unavailable data. Ultimately, according to the inclusion and exclusion criteria, 14 studies met the criteria for systematic review, and 9 studies were eligible for meta-analysis. The screening process is illustrated in the flow diagram in Figure 1.

**Figure 1:**
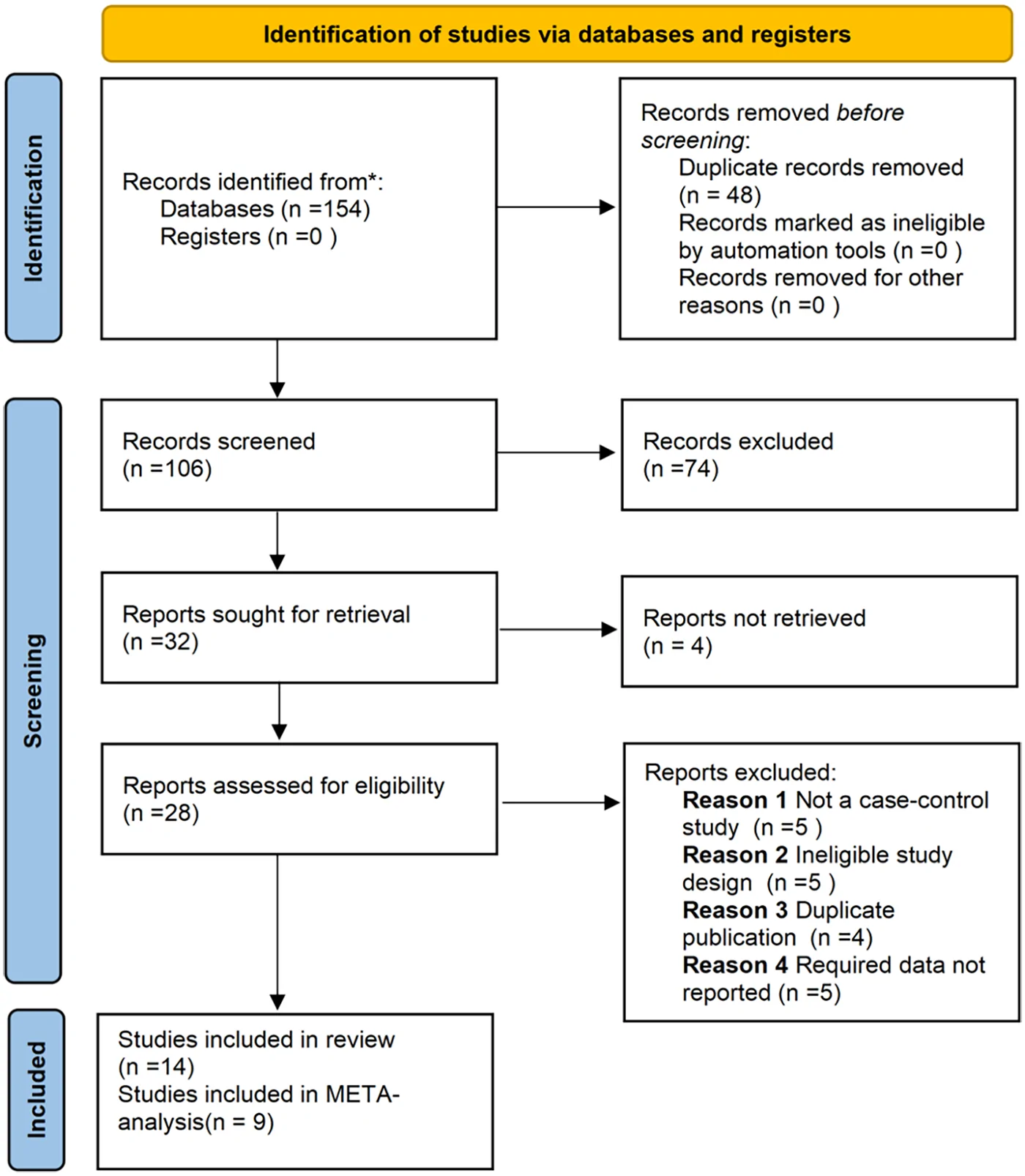
PRISMA flow diagram of the study selection process. A total of 154 records were identified from PubMed, Embase, Cochrane, and Web of Science. After removing 48 duplicates, 106 records were screened: 74 records were excluded based on their titles and abstracts; 32 full-text articles were assessed for eligibility, of which 18 were excluded for various reasons (full text not available, non-case–control design, ineligible exposure/outcome, duplicate publications, or unavailable data). Finally, 14 studies were included in the systematic review, and 9 studies were eligible for the meta-analysis.

#### 2.2.2 Study characteristics

Fourteen studies (Table 1) were included in this study, all of which were case–control or nested case–control studies published between 2016 and 2025. The sample size of the included studies ranged from 83 to 2,181, with case group sample sizes ranging from 44 to 953 and control group sample sizes ranging from 44 to 1,273. All studies reported an association between plasma TMAO levels and ASCVD events. Geographic distribution: The included studies covered multiple countries and regions, among which seven studies were conducted in China (12–18), four studies were conducted in the United States (19–22), one study was conducted in Europe (23), one study was conducted in Turkey (24), and one multi-country collaborative study [(25), included the US and Chinese populations].

**Table 1:** Characteristics of the included studies.

Study	Study design	Region	Cases (*n*)	Controls (*n*)	Outcome definition	TMAO assay	Effect size type	NOS score
Chen et al. (12)	Case–control	China	291	235	LAA ischemic stroke	LC-MS	Continuous	6
Heianza et al. (19)	Nested case–control	United States	380	380	CHD	HPLC-ESI-MS/MS	T3 vs. T1	8
Tascanov et al. (24)	Case–control	Turkey	44 (young) + 39 (elderly)	44	ACS	ELISA	Continuous	5
Sun et al. (13)	Case–control	China	429	429	CHD	LC-MS/MS	Continuous	7
Sun et al. (14)	Case–control	China	953	953	Ischemic stroke	HPLC-MS/MS	Continuous + (Q4 vs. Q1)	7
Liu et al. (15)	Nested case–control	China	412	412	Ischemic stroke	UHPLC-MS/MS	Continuous + (Q4 vs. Q1)	8
Yu et al. (25)	Multi-stage study	United States + China	864	864	CHD	UHPLC-MS/MS	Continuous	8
Dai et al. (16)	Case–control	China	254	247	ACS	UHPLC-MS/MS	Continuous + (Q4 vs. Q1)	6
Xu et al. (17)	Case–control	China	81	51	LAA/CE stroke	LC-MS/MS	Continuous	6
Gencer et al. (20)	Nested case–control	Europe/United States	597	1,206	MACE	LC-MS/MS	Continuous + (Q4 vs. Q1)	9
Liu et al. (21)	Nested case–control	United States	608	608	CAD	HPLC-ESI-MS/MS	Continuous + (T3 vs. T1)	8
Tang et al. (23)	Nested case–control	United Kingdom	908	1,273	CAD	LC-MS/MS	Q4 vs. Q1	8
Dong et al. (18)	Case–control	China	184	114	UAP	LC-MS/MS	Continuous	5
Talmor-Barkan et al. (22)	Nested case–control	United States	88	273	Hard ASCVD	LC-MS/MS	Q4 vs. Q1	8

ACS, acute coronary syndrome; ASCVD, atherosclerotic cardiovascular disease; CAD, coronary artery disease; CHD, coronary heart disease; CI, confidence interval; ELISA, enzyme-linked immunosorbent assay; HPLC, high-performance liquid chromatography; LC-MS, liquid chromatography–mass spectrometry; MACE, major adverse cardiovascular events; NOS, Newcastle–Ottawa Scale; Q, quartile; T, tertile; TMAO, trimethylamine N-oxide; UHPLC, ultra-high-performance liquid chromatography.

For the study design, among the included studies, six were nested case–control studies (15, 19–23) and one had a mixed/combined study design (25), seven were case–control studies (12–14, 16–18, 24).

Outcome definition: Study outcomes included CAD, ACS, UAP, MACE, ischemic stroke, large artery atherosclerotic stroke (LAA), cardioembolic stroke (CE), and hard ASCVD events.

TMAO detection methods: All studies used liquid chromatography-tandem mass spectrometry (LC-MS/MS, HPLC-MS/MS, UHPLC-MS/MS, or HPLC-ESI-MS/MS) to measure plasma TMAO levels, except for one study that used ELISA (24).

Effect size types: Among the studies included in the meta-analysis, seven reported categorical effect sizes (highest quantile vs. lowest quantile), eight reported continuous effect sizes (per 1-SD increase or per 1-unit increase), and five studies reported both types of effect sizes.

Quality assessment: The methodological quality of the included studies was assessed using the Newcastle–Ottawa Scale (NOS). The NOS scores ranged from 5 to 9, with nine studies (64.3%) scoring 7 or higher. The specific score distribution was as follows: one study scored 9, six studies scored 8, two studies scored 7, three studies scored 6, and two studies scored 5.

#### 2.2.3 Association between plasma TMAO levels and atherosclerotic cardiovascular disease

Based on the effect size types reported in the included studies, the studies were divided into two categories for pooled analysis: categorical effect sizes (highest quantile vs. lowest quantile) and continuous effect sizes (per 1-SD increase or per 1-unit increase). The details are presented in Table 2.

**Table 2:** Effect estimates and subgroups in the meta-analysis.

Categorical effect size				
Study	OR (95% CI)	Region^a^	Study design	Population type^b^
Sun et al. (14)	1.81 (1.27–2.59)	China	Case–control	Primary prevention
Liu et al. (15)	1.81 (1.14–2.86)	China	Nested case–control	Primary prevention
Dai et al. (16)	1.09 (0.64–1.85)	China	Case–control	Secondary prevention
Gencer et al. (20)	1.10 (0.79–1.55)	Western	Nested case–control	Secondary prevention
Liu et al. (21)	1.23 (0.89–1.70)	Western	Nested case–control	Primary prevention
Tang et al. (23)	1.41 (1.07–1.85)	Western	Nested case–control	Primary prevention
Talmor-Barkan et al. (22)	2.77 (1.05–7.70)	Western	Nested case–control	Primary prevention

Continuous effect size (SMD)				
Study	SMD (95% CI)	Region^a^	Study design	Population type^b^
Sun et al. (14)	−0.07 (−0.25–0.10)	China	Case–control	Primary prevention
Liu et al. (15)	1.11 (0.86–1.36)	China	Nested case–control	Primary prevention
Dai et al. (16)	0.27 (0.18–0.36)	China	Case–control	Secondary prevention
Xu et al. (17)	0.32 (0.19–0.46)	China	Case–control	Secondary prevention
Dong et al. (18)	0.59 (0.23–0.95)	China	Case–control	Secondary prevention

a Region: “Western” includes studies from the United States (20–22), Europe (20), and the United Kingdom (23). “Multiple regions” indicates that the study included participants from both the United States and China (25). All other studies were conducted exclusively in China.

b Population Type: “Primary prevention” = no prior cardiovascular disease at baseline; “Secondary prevention” = established cardiovascular disease at baseline.

Seven studies (14–16, 20–23) reported categorical effect sizes for plasma TMAO (highest quantile vs. lowest quantile), with a total sample size of 8,792 participants (3,820 cases and 4,972 controls). Heterogeneity among the studies was low (*I*^2^ = 28.8%, *P* = 0.208), and a fixed-effect model was used for the pooled analysis. The meta-analysis results showed that high plasma TMAO levels were significantly associated with ASCVD risk, with a pooled OR of 1.39 (95% CI: 1.21–1.61, *P* < 0.001). The effect sizes of the individual studies ranged from 1.09 to 2.77, among which Talmor-Barkan et al. (22) reported the highest effect size (OR = 2.77, 95% CI: 1.05–7.70) and Dai et al. (16) reported the lowest effect size (OR = 1.09, 95% CI: 0.64–1.85). The weight distribution of each study was relatively balanced, with Tang et al. (23) reporting the highest weight (27.31%) and Talmor-Barkan et al. (22) reporting the lowest weight (2.06%). No single study had a decisive influence on the pooled effect size. The forest plot is shown in Figure 2.

**Figure 2:**
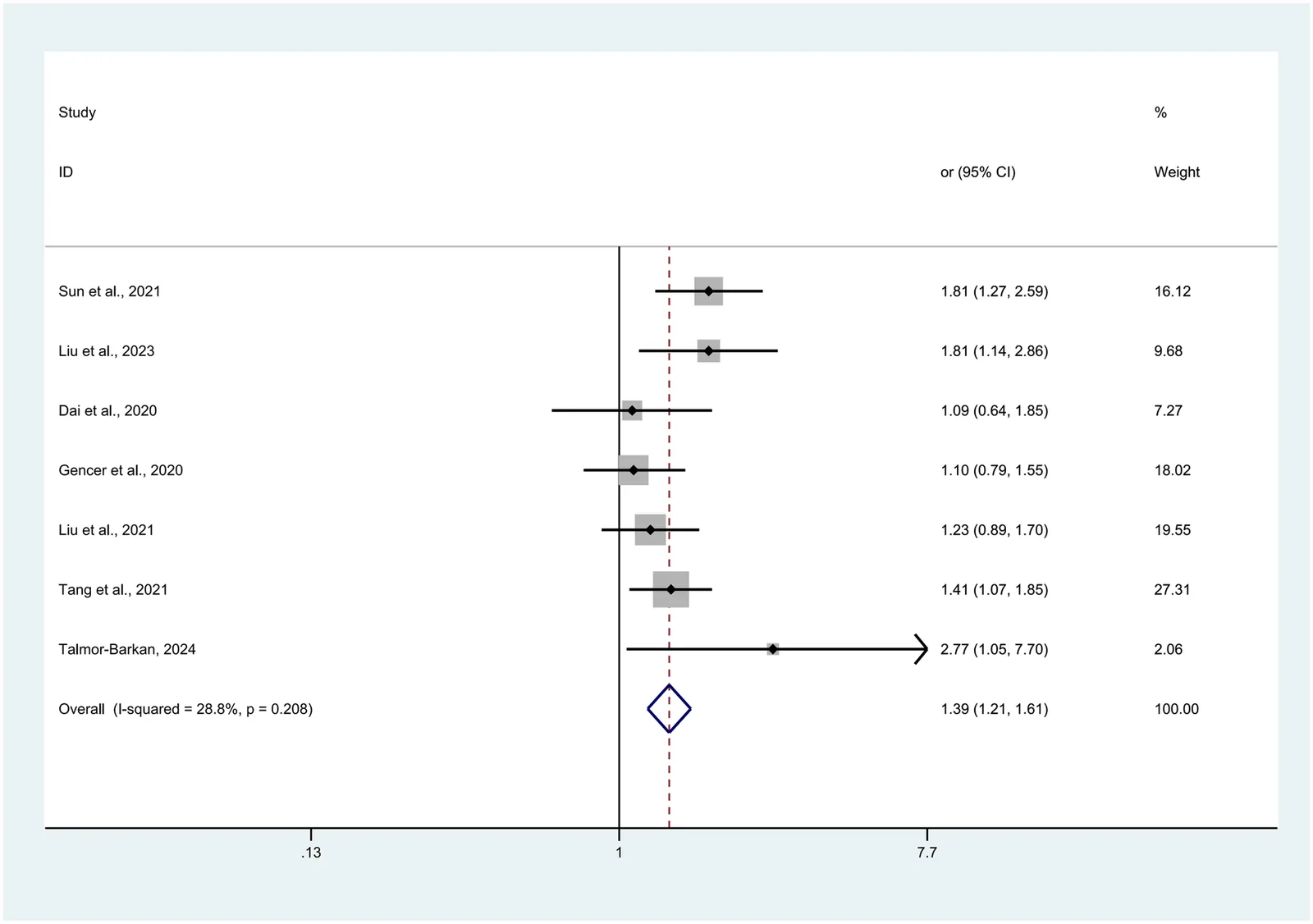
Forest plot (categorical variables). Squares represent ORs with sizes proportional to study weight; horizontal lines indicate 95% CIs; the diamond represents the pooled OR from the fixed-effect model (*I*^2^ = 28.8%, *P* = 0.208). The overall OR was 1.39 (95% CI: 1.21–1.61, *P* < 0.001). The highest versus lowest quantile comparison was defined as quartile 4 versus quartile 1 (14–16, 20, 22, 23) and tertile 3 versus tertile 1 (19, 21). All ORs were extracted from the most fully adjusted models for traditional cardiovascular risk factors, as reported in each original study.

Eight studies (14–18, 20, 21, 25) reported continuous effect sizes for plasma TMAO (per 1-unit increase), with a total sample size of 8,408 participants (3,953 cases and 4,455 controls). According to the prespecified analysis strategy, we adopted the SMD as a common effect measure. Five studies (14–18) provided data that could be converted into SMD for pooling, with a total sample size of 1,763 participants (932 cases and 831 controls). The analysis showed substantial heterogeneity among the studies (*I*^2^ = 93.4%, *P* < 0.001); therefore, a random-effects model was used for the pooled analysis. The meta-analysis results showed that TMAO levels were significantly associated with ASCVD risk, with a pooled SMD of 0.43 (95% CI: 0.14–0.71). The effect sizes of the individual studies ranged from −0.07 to 1.11, among which Dong et al. (18, 19) reported the highest effect size (SMD = 1.11, 95% CI: 0.86–1.36), and Dai et al. (16) reported the lowest effect size (SMD = −0.07, 95% CI: −0.25–0.10), with the latter showing an opposite direction of effect compared with the other studies. The weight distribution of each study showed some variation, with Sun et al. (14) (21.97%), Liu et al. (15) (21.40%), and Dai et al. (16) (20.74%) reporting relatively higher weights, while Xu et al. (17) (16.67%) and Dong et al. (18) (19.23%) reported relatively lower weights (Figure 3).

**Figure 3:**
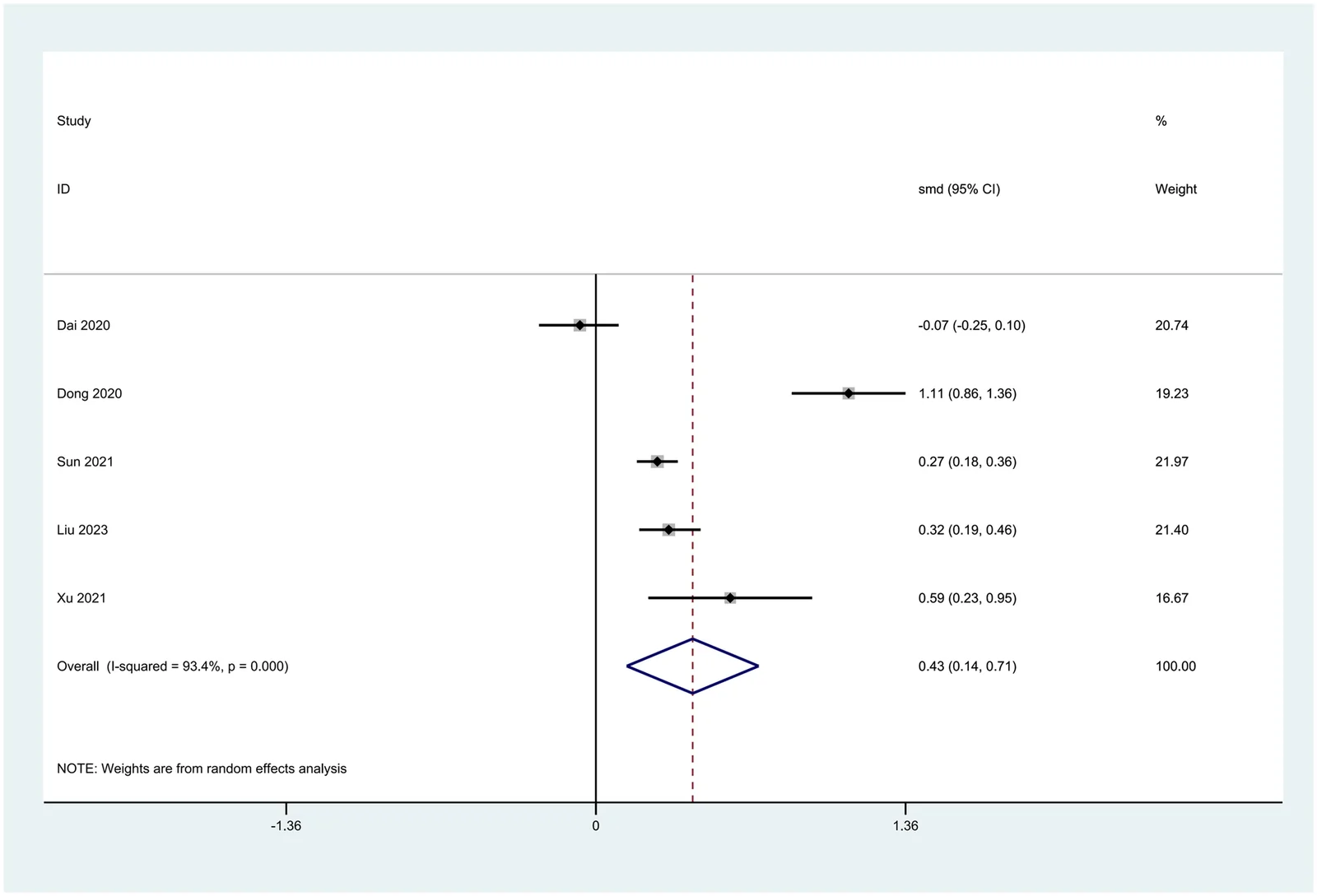
Forest plot (continuous variables). Random-effects pooled SMD = 0.43 (95% CI: 0.14–0.71); heterogeneity: *I*^2^ = 93.4%, *P* < 0.001. Studies included: Dai et al. (16), Dong et al. (18), Sun et al. (14), Liu et al. (15), and Xu et al. (17).

#### 2.2.4 Subgroup analysis

 Subgroup analyses were performed based on region, population type, and study design to explore the potential sources of heterogeneity. Other prespecified subgroup analyses were not conducted because of an insufficient number of studies.

##### 2.2.4.1 Categorical variable analysis

In the categorical variable analysis, subgroup analysis by region (Figure 4) showed that the pooled OR for the Chinese population subgroup (three studies) was 1.62 (95% CI: 1.26–2.08), with low heterogeneity (*I*^2^ = 26.9%, *P* = 0.255). The pooled OR for the Western population subgroup (four studies) was 1.29 (95% CI: 1.09–1.54), with low heterogeneity (*I*^2^ = 16.8%, *P* = 0.307). The difference between the subgroups was not statistically significant (*P* for interaction = 0.148), suggesting that the association between TMAO and ASCVD was consistent across different regional populations.

**Figure 4:**
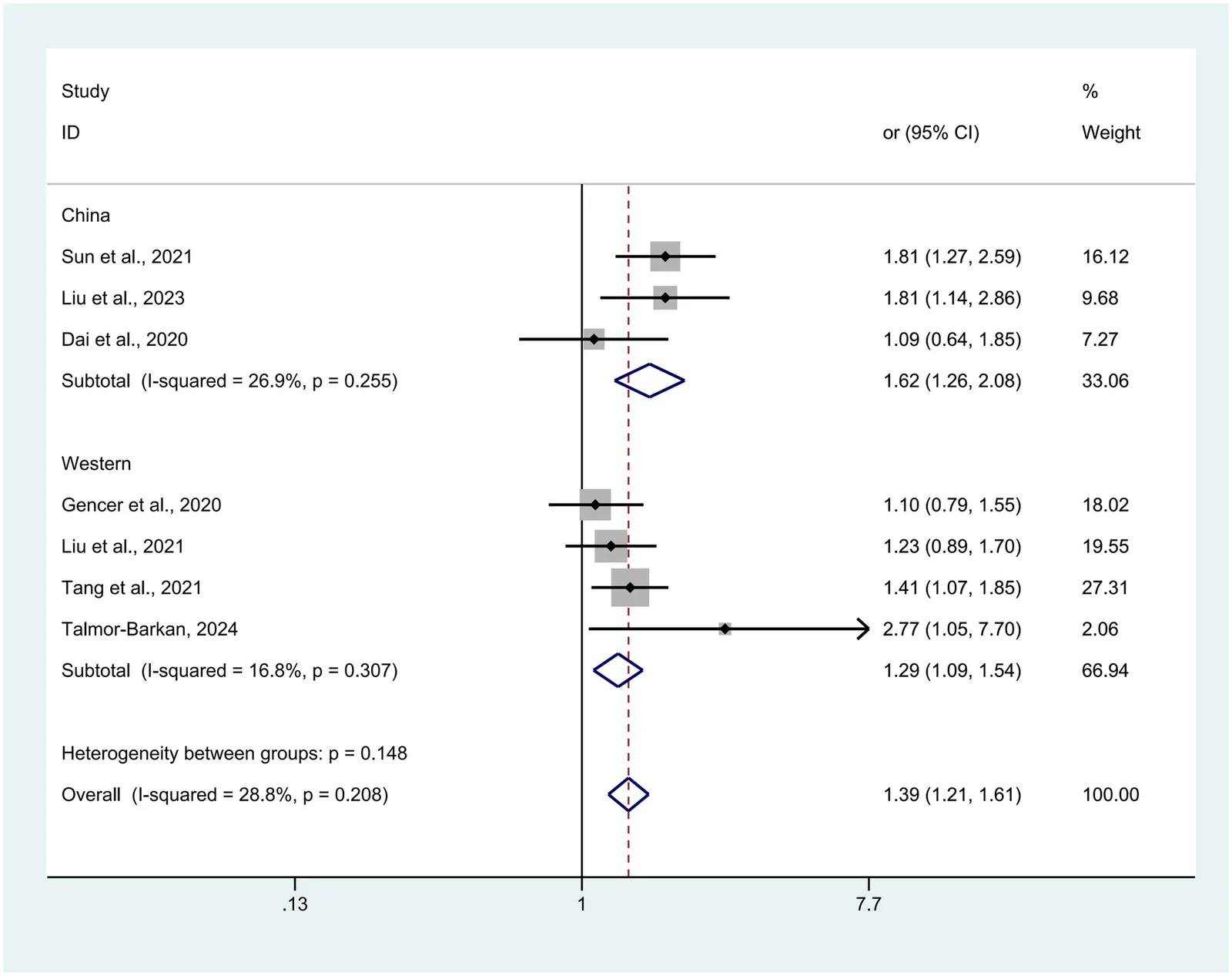
Subgroup analysis by geographic region (categorical variables). The pooled OR for the Chinese population subgroup (three studies) was 1.62 (95% CI: 1.26–2.08), with low heterogeneity (*I*^2^ = 26.9%). The pooled OR for the Western population subgroup (four studies) was 1.29 (95% CI: 1.09–1.54), with low heterogeneity (*I*^2^ = 16.8%). The differences between the subgroups were not statistically significant (*P* for interaction = 0.148). The quantile definitions and adjustment details for each study are the same as those described in the legend of Figure 2.

Subgroup analysis by the population type (Figure 5) showed that the pooled OR for the primary prevention population (no history of ASCVD at baseline, five studies) was 1.51 (95% CI: 1.28–1.78), with low heterogeneity (*I*^2^ = 16.6%, *P* = 0.309). The pooled OR for the secondary prevention population (history of ASCVD at baseline, two studies) was 1.10 (95% CI: 0.83–1.46), with no heterogeneity (*I*^2^ = 0.0%, *P* = 0.977). The difference between the subgroups was marginally significant (*P* for interaction = 0.057), suggesting that the association between TMAO and ASCVD may be stronger in the primary prevention population.

**Figure 5:**
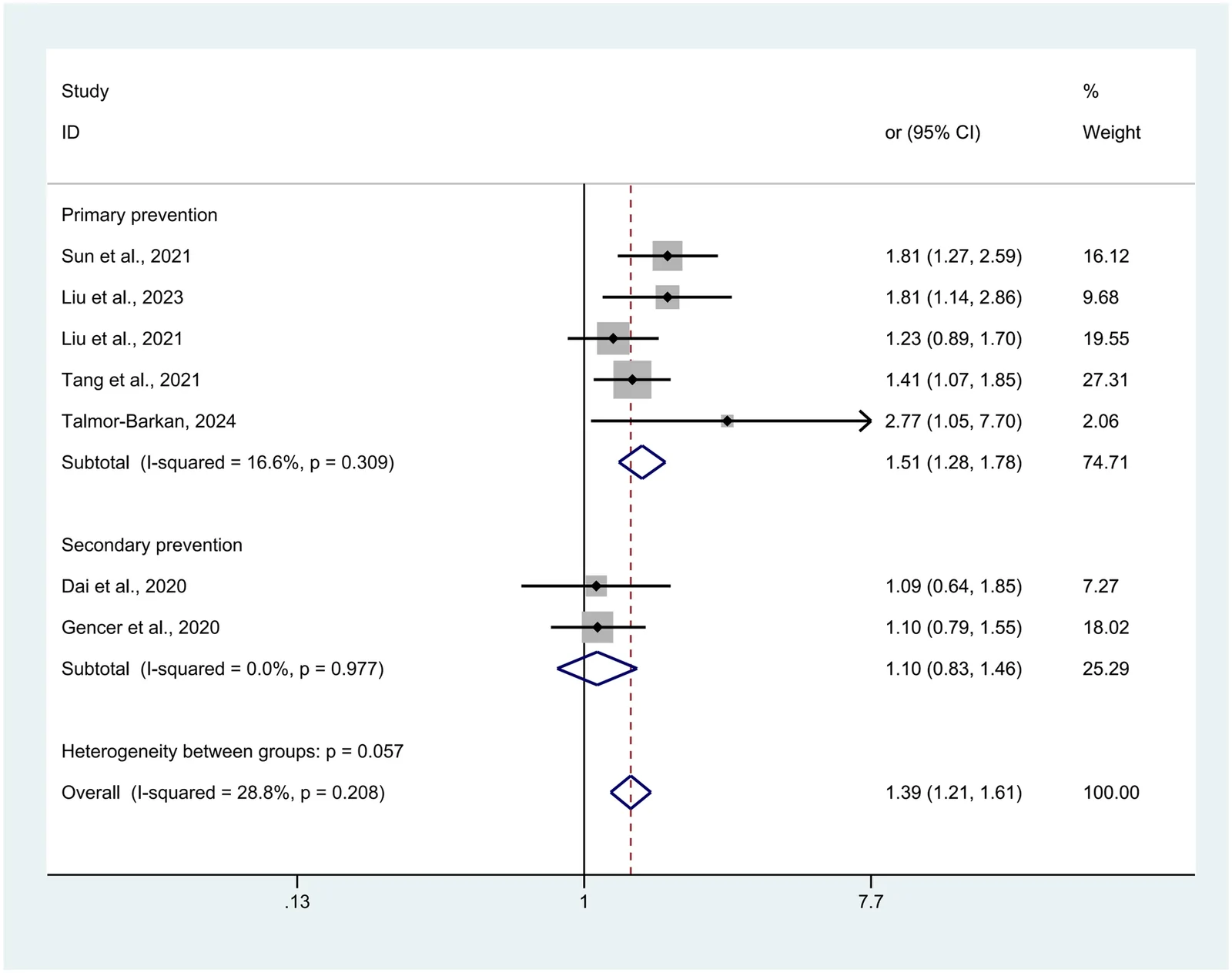
Subgroup analysis by study population type (categorical variables). The pooled OR for the primary prevention population (five studies) was 1.51 (95% CI: 1.28–1.78), with low heterogeneity (*I*^2^ = 16.6%). The pooled OR for the secondary prevention population (two studies) was 1.10 (95% CI: 0.83–1.46), with no heterogeneity (*I*^2^ = 0.0%). The differences between the subgroups were marginally significant (*P* for interaction = 0.057). The quantile definitions and adjustment details for each study are the same as those described in the legend of Figure 2.

Subgroup analysis by study design (Figure 6) showed that the pooled OR for retrospective case–control studies (two studies) was 1.55 (95% CI: 1.15–2.08), with moderate heterogeneity (*I*^2^ = 58.6%, *P* = 0.120). The pooled OR for nested case–control studies (5 studies) was 1.35 (95% CI: 1.15–1.59), with low heterogeneity (*I*^2^ = 25.9%, *P* = 0.249). The difference between the subgroups was not statistically significant (*P* for interaction = 0.431), indicating that the study design did not significantly influence the results. Overall, the subgroup analysis results supported the robustness of the main findings.

**Figure 6:**
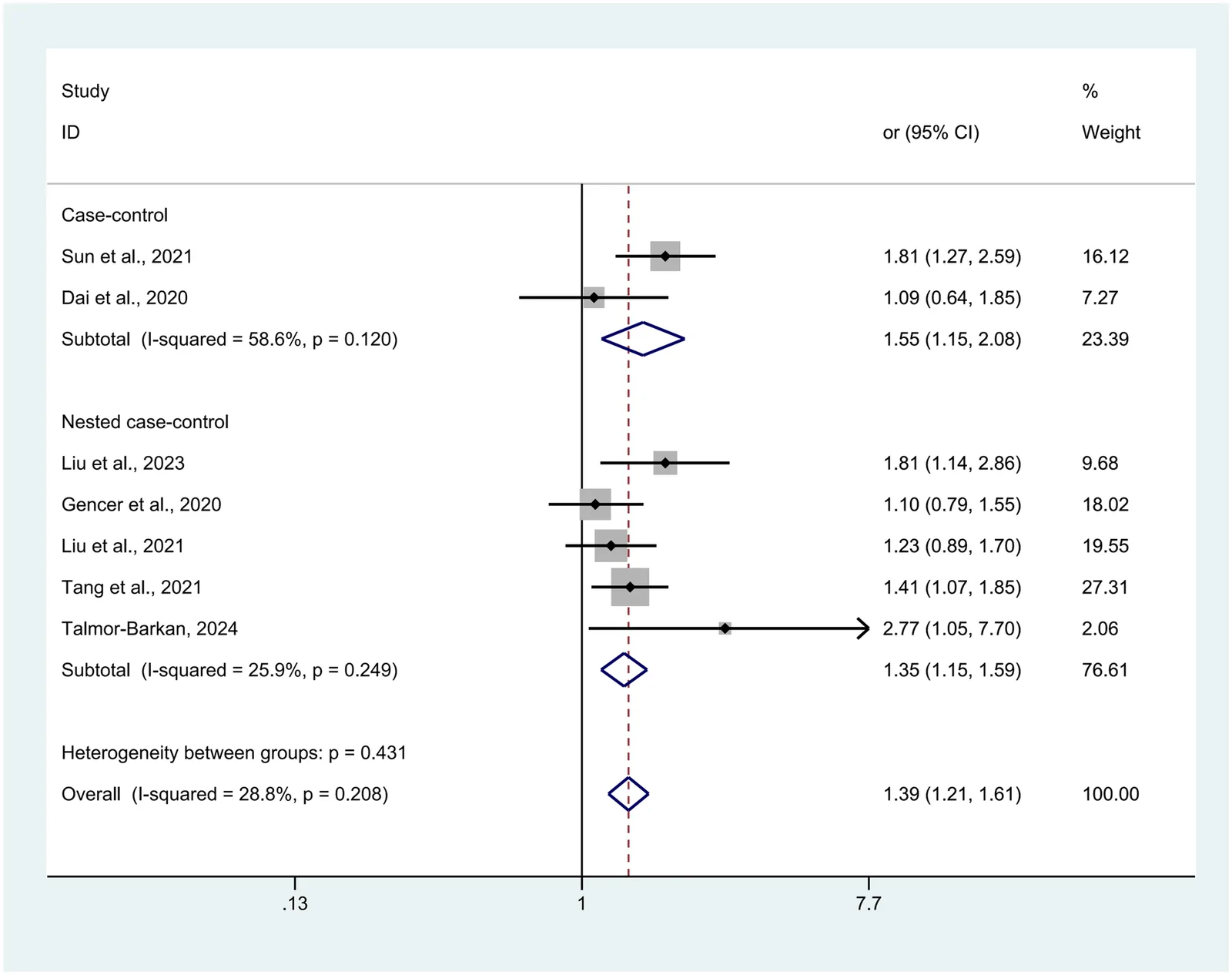
Subgroup analysis by study design (categorical variable). The pooled OR for retrospective case-control studies (two studies) was 1.55 (95% CI: 1.15–2.08), with moderate heterogeneity (*I*^2^ = 58.6%). The pooled OR for nested case-control studies (five studies) was 1.35 (95% CI: 1.15–1.59), with low heterogeneity (*I*^2^ = 25.9%). The difference between subgroups was not statistically significant (*P* for interaction = 0.431). The quantile definitions and adjustment details for each study are the same as those described in the legend of Figure 2.

##### 2.2.4.2 Continuous variable analysis

In the continuous variable analysis, subgroup analyses were performed descriptively to explore potential sources of substantial heterogeneity (*I*^2^ = 93.4%), given the limited number of included studies (*n* = 5). With respect to population type (Figure 7), the primary prevention subgroup (2 studies) demonstrated a small but consistent positive association (SMD = 0.29, 95% CI: 0.21–0.36, *I*^2^ = 0.0%), while the secondary prevention subgroup (three studies) showed marked instability (SMD = 0.54, 95% CI: −0.25–1.33, *I*^2^ = 96.6%). Stratification by study design (Figure 8) revealed that the single nested case–control study (15) yielded a precise estimate (SMD = 0.32, 95% CI: 0.19–0.46), whereas the four case–control studies exhibited high heterogeneity (*I*^2^ = 95.0%). No subgroup analysis by region was conducted because all the included studies were from China. Owing to the small number of studies per subgroup, these findings should be interpreted as hypothesis-generating, and no formal tests for subgroup differences were performed.

**Figure 7:**
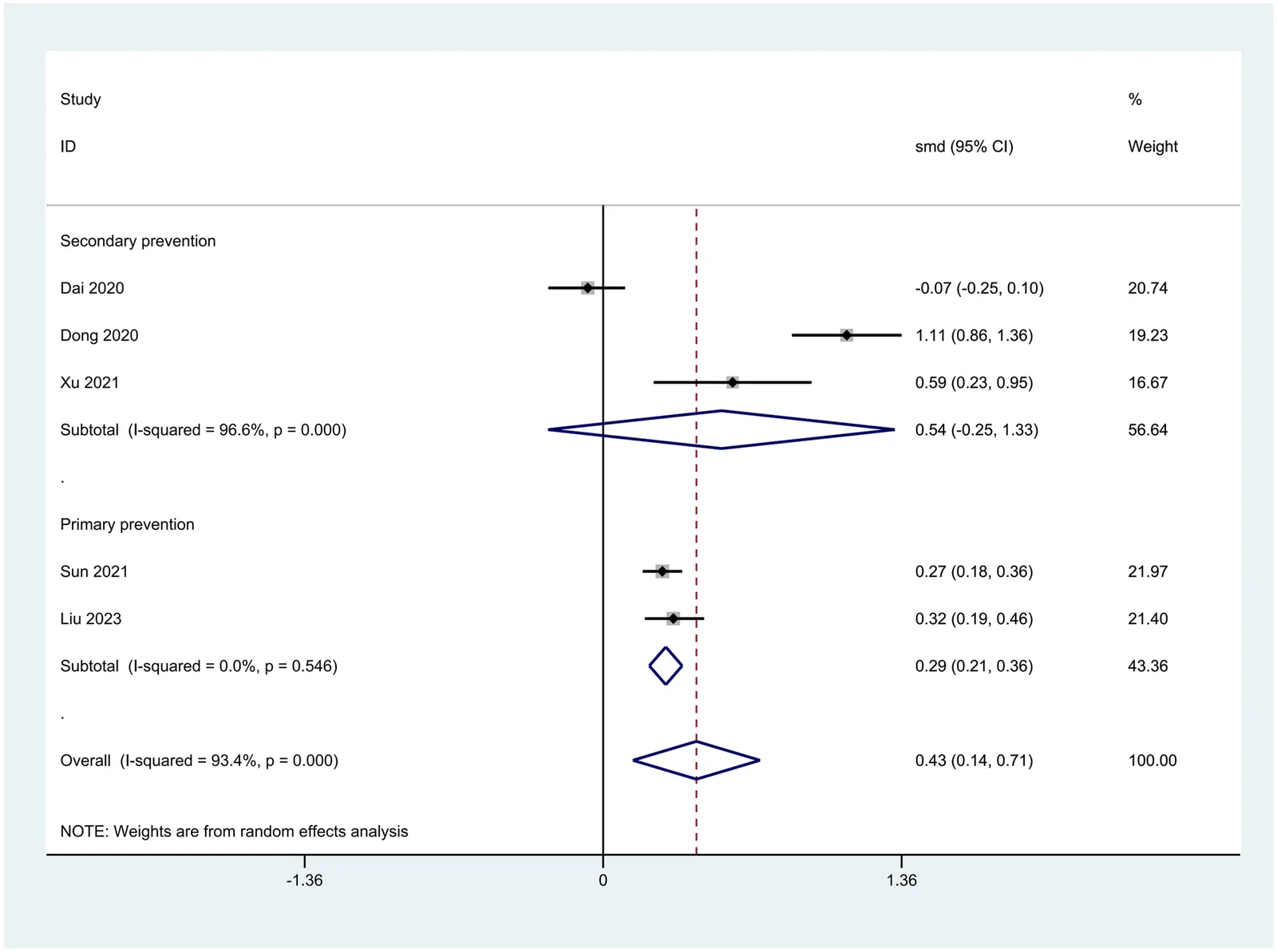
Subgroup analysis by population type (continuous variables). Subgroup analysis by population type. Random-effects model: secondary prevention subgroup (*n* = 3 studies) SMD = 0.54 (95% CI: −0.25 to 1.33), *I*^2^ = 96.6%; primary prevention subgroup (*n* = 2 studies) SMD = 0.29 (95% CI: 0.21–0.36), *I*^2^ = 0.0%; overall SMD = 0.43 (95% CI: 0.14–0.71), *I*^2^ = 93.4%.

**Figure 8:**
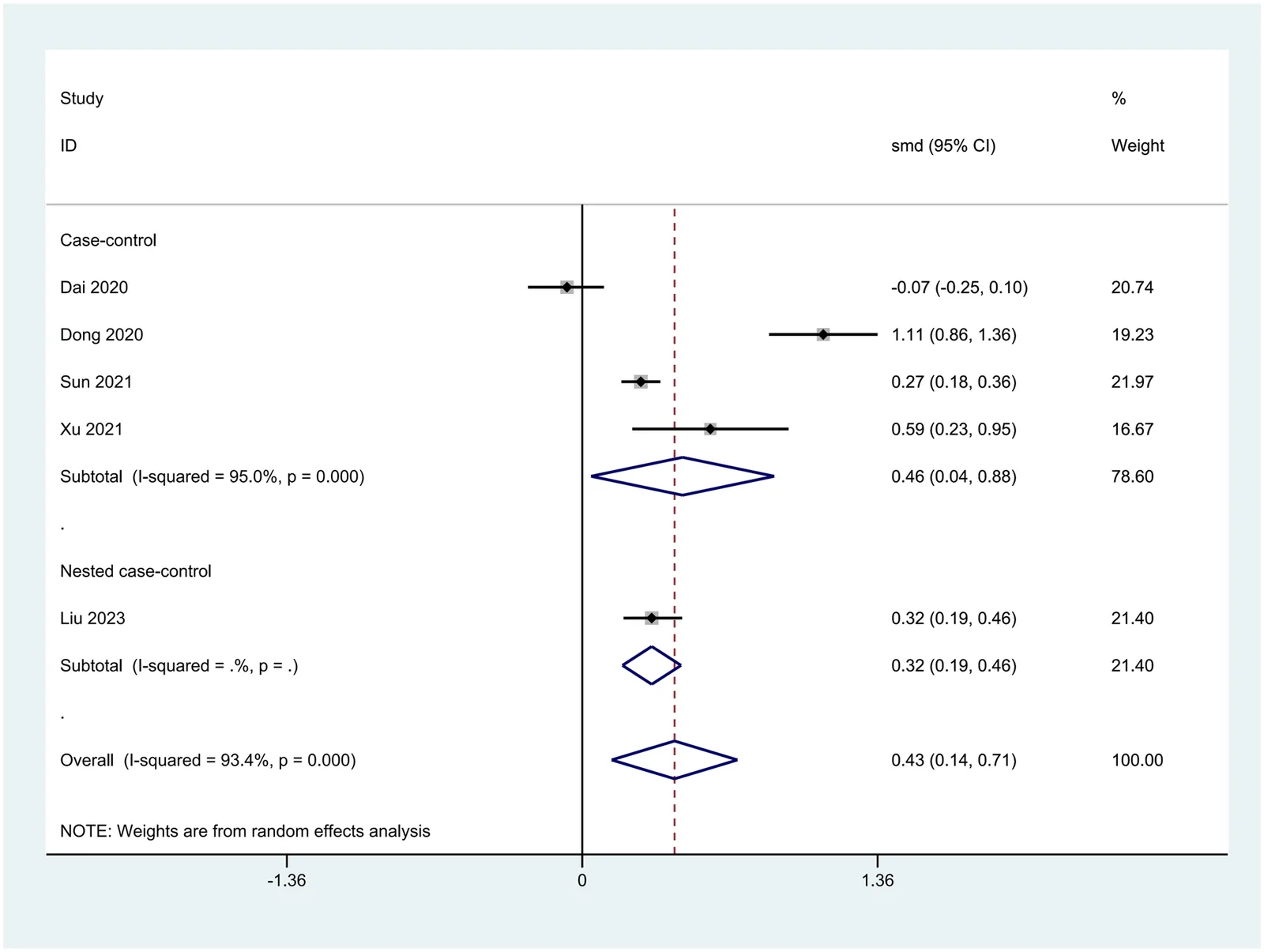
Subgroup analysis by study design (continuous variables). Subgroup analysis by study design. Case–control subgroup (*n* = 4): heterogeneity *I*^2^ = 95.0%; nested case–control (*n* = 1): single study.

#### 2.2.5 Sensitivity analysis

To assess the robustness of the meta-analysis results, we primarily performed a sensitivity analysis using the leave-one-out method, recalculating the pooled effect size after sequentially removing each study.

##### 2.2.5.1 Categorical variable analysis

The leave-one-out sensitivity analysis (Figure 9) showed that after sequentially removing each study, the pooled OR values ranged from 1.21 to 1.29, all of which were within the 95% confidence interval of the overall pooled effect size (OR = 1.39, 95% CI: 1.21–1.61). The pooled OR reached its lowest point after removing Sun et al. (14) (OR = 1.21, 95% CI: 1.13–1.39). After removing Liu et al. (15) and Dai et al. (16), the pooled OR was 1.29 (95% CI: 1.21–1.31). All leave-one-out analysis results remained statistically significant, and the effect size did not change direction, indicating that no single study had a decisive influence on the overall pooled effect size. Thus, the results of the meta-analysis of categorical variables were relatively robust.

**Figure 9:**
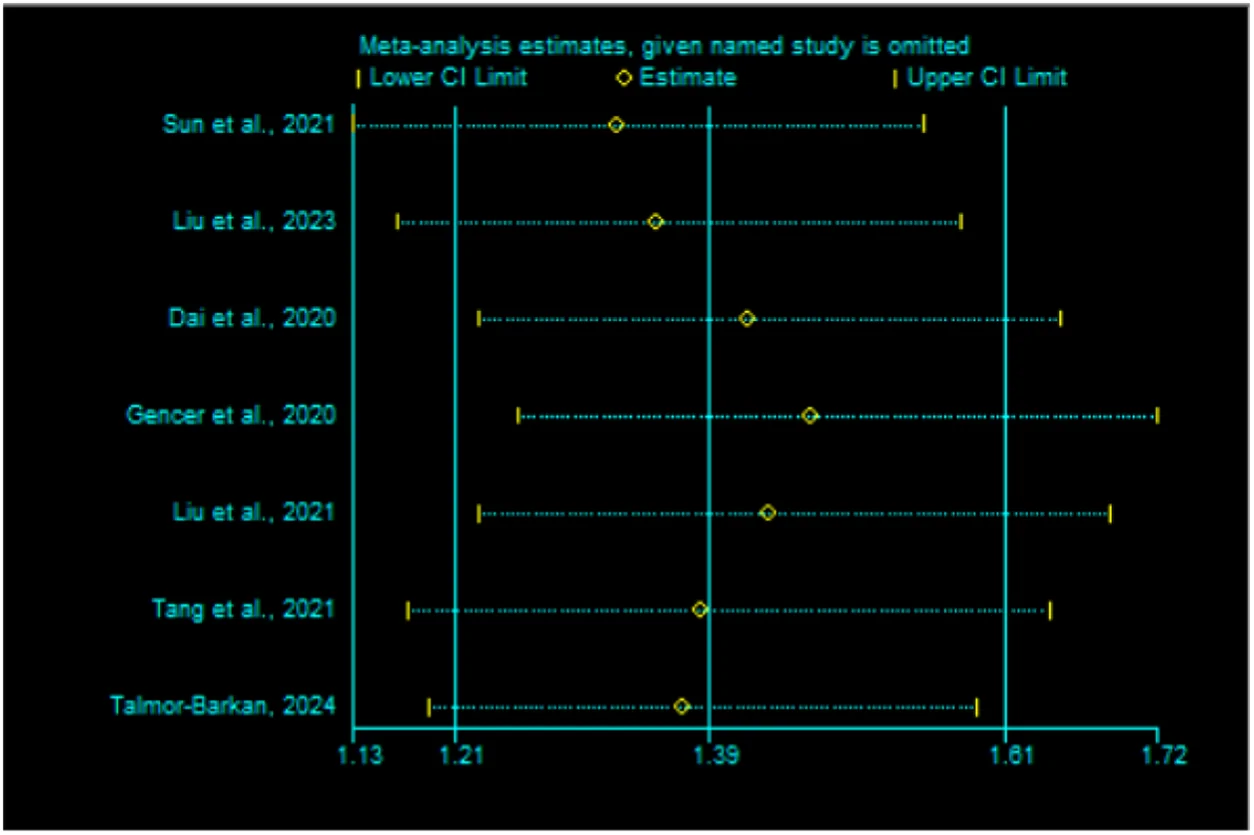
Sensitivity analysis of categorical variables using the leave-one-out method. The pooled OR values after sequentially removing each study ranged from 1.21 to 1.29, all within the 95% confidence interval of the overall pooled effect size (OR = 1.39, 95% CI: 1.21–1.61). No single study had a decisive influence on the overall effect, indicating robust results.

##### 2.2.5.2 Continuous variable analysis

The leave-one-out sensitivity analysis (Figure 10) showed that after sequentially removing each study, the pooled SMD values ranged from 0.36 to 0.56. After removing Dong et al. (18) (SMD = 1.11, a study with high effect size and potential bias, NOS = 5), the pooled SMD decreased from 0.43 to 0.36 (95% CI: 0.24–0.48), and the heterogeneity decreased substantially from 93.4% in the original analysis to 40.2% (*P* for heterogeneity = 0.17), suggesting that this extreme value was a major source of the observed heterogeneity. After removing Dai et al. (16) (SMD = −0.07, the only study showing an opposite direction of effect), the pooled SMD increased to 0.56 (95% CI: 0.35–0.77), with heterogeneity remaining high (*I*^2^ = 85.6%), indicating that this study had a diluting effect on the overall effect size. After removing Sun et al. (14), Liu et al. (15), and Xu et al. (17) individually, the pooled SMD remained relatively stable, ranging from 0.41 to 0.46, with little change in heterogeneity. Overall, the leave-one-out analysis indicated that the results of the continuous variable meta-analysis (SMD = 0.43, 95% CI: 0.14–0.71) were influenced by the extreme values of Dong et al. (18) and Dai et al. (16). After excluding these two influential studies, the pooled SMD was 0.36 (95% CI: 0.24–0.48) with acceptable heterogeneity (*I*^2^ = 40.2%), supporting a modest but robust positive association between TMAO levels and ASCVD risk.

**Figure 10:**
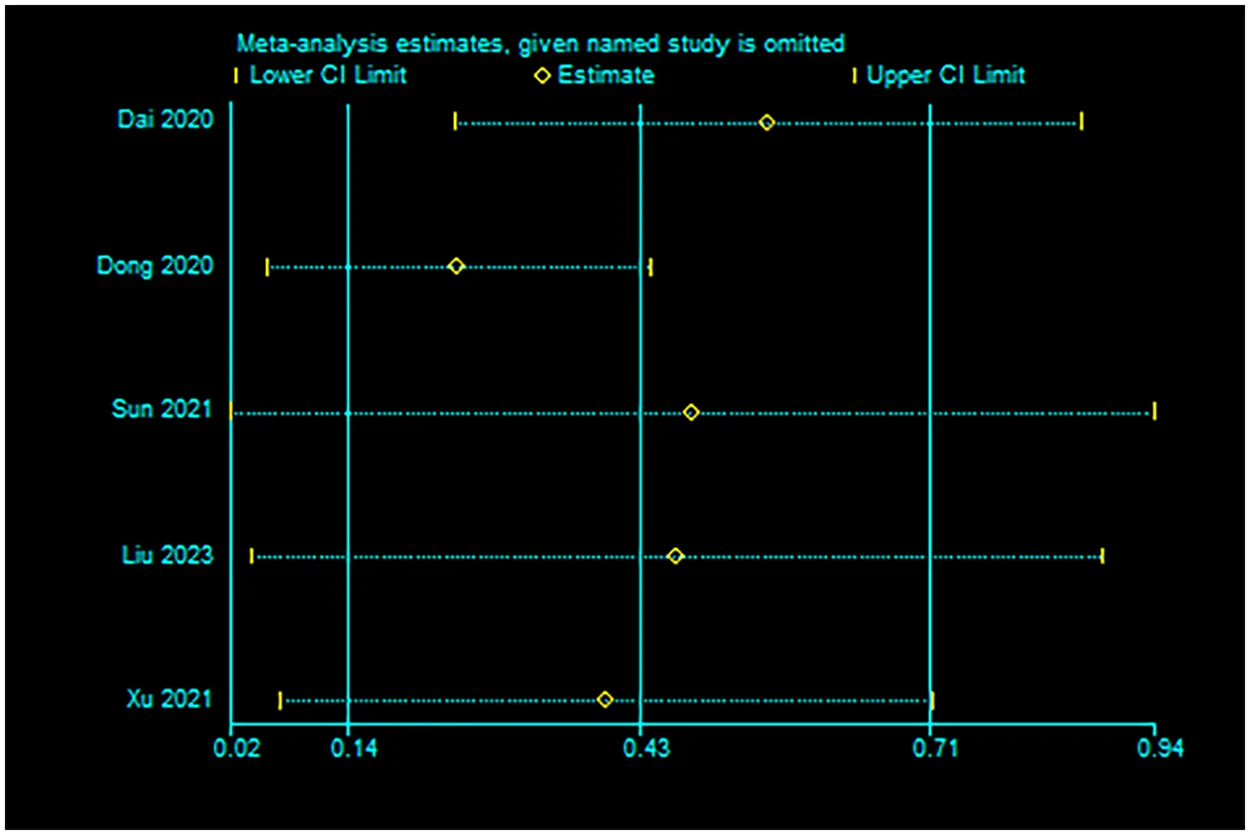
Sensitivity analysis of continuous variables using the leave-one-out method. Sensitivity analysis of continuous variables. The leave-one-out meta-analysis showed that the omission of any single study (14–18) did not substantially alter the pooled SMD (range: 0.43–0.43) or its 95% CI (0.14–0.71), confirming the robustness of the overall estimate.

#### 2.2.6 Publication bias

To assess publication bias in the meta-analysis results, we used funnel plots for visual assessment and supplemented them with Egger’s regression test for quantitative evaluation.

##### 2.2.6.1 Categorical variable analysis

In the categorical variable analysis, the funnel plot (Figure 11) showed that the 7 study points were basically symmetrically distributed on both sides of the pooled effect size, with most studies located at the top of the funnel plot, and no obvious asymmetry was observed. Owing to the small number of included studies (*n* = 7), the statistical power for assessing funnel plot symmetry was limited and was used only as a qualitative reference. Egger's regression plot (Figure 12) further showed that the study points were evenly distributed on both sides of the regression line, which was nearly horizontal. Egger's regression test did not indicate significant publication bias (*t* = 0.64, *P* = 0.545). Considering both the funnel plot symmetry and statistical test results, no significant publication bias was detected in this study. However, because of the small number of included studies (*n* = 7), the statistical power of the publication bias test was limited, and the results should be interpreted with caution, as the possibility of publication bias cannot be completely ruled out.

**Figure 11:**
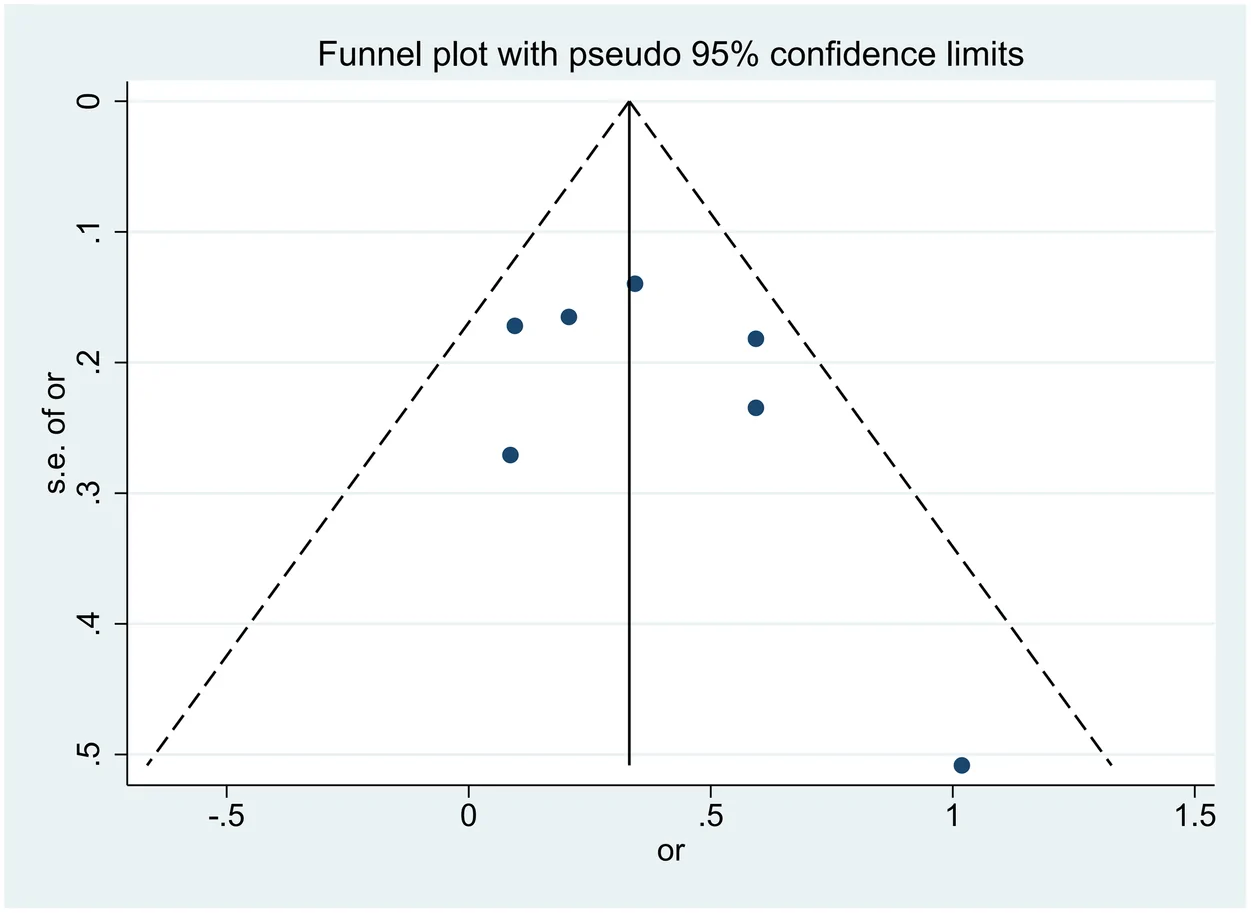
Funnel plot for assessing publication bias in the categorical variable analysis (seven studies). The study points were symmetrically distributed around the pooled effect size, with no obvious asymmetry. Egger's regression test did not indicate significant publication bias (*t* = 0.64, *P* = 0.545).

**Figure 12:**
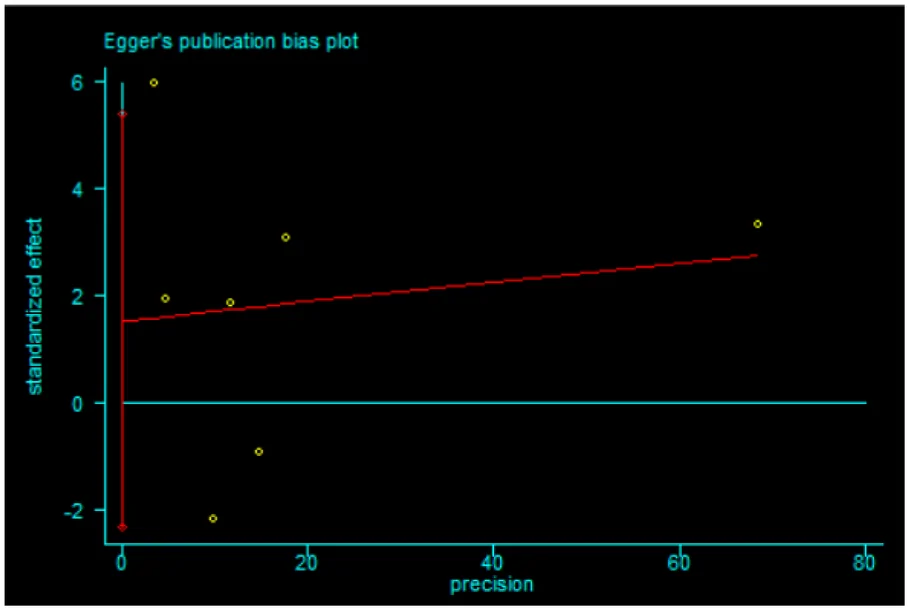
Egger's regression plot for publication bias in the categorical variable analysis. The study points were evenly distributed on both sides of the regression line, which was nearly horizontal, confirming no significant publication bias (*t* = 0.64, *P* = 0.545).

##### 2.2.6.2 Continuous variable analysis

In the continuous variable analysis, the funnel plot (Figure 13) showed that the five study points were relatively symmetrically distributed on both sides of the pooled effect size, with no obvious asymmetry observed. Owing to the small number of included studies (*n* = 5), the statistical power for assessing funnel plot symmetry was severely limited and was used only as a qualitative reference. Egger's regression test (Figure 14) did not indicate significant publication bias (*t* = −0.82, *P* = 0.474). Considering both the funnel plot symmetry and statistical test results, no significant publication bias was detected in this study. However, because of the very small number of included studies (*n* = 5), the statistical power of the publication bias test was extremely limited, and the results should be interpreted with great caution, as the possibility of publication bias cannot be completely ruled out.

**Figure 13:**
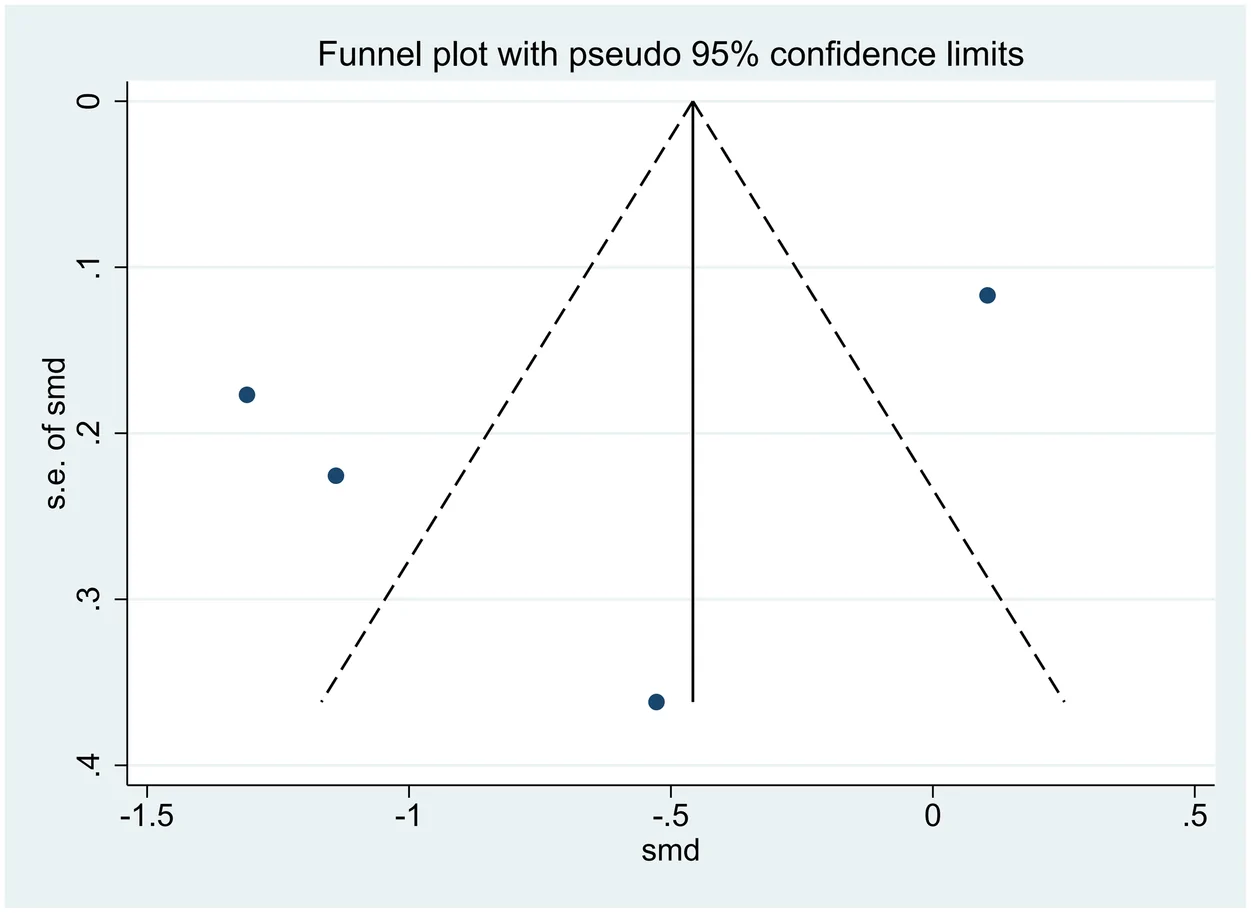
Funnel plot for assessing publication bias in the continuous variable analysis (five studies). Funnel plot with pseudo 95% confidence limits. The studies were symmetrically distributed around the pooled SMD (0.43, 95% CI: 0.14–0.71). Egger's test suggested no significant publication bias (*t* = 1.08, *P* = 0.323).

**Figure 14:**
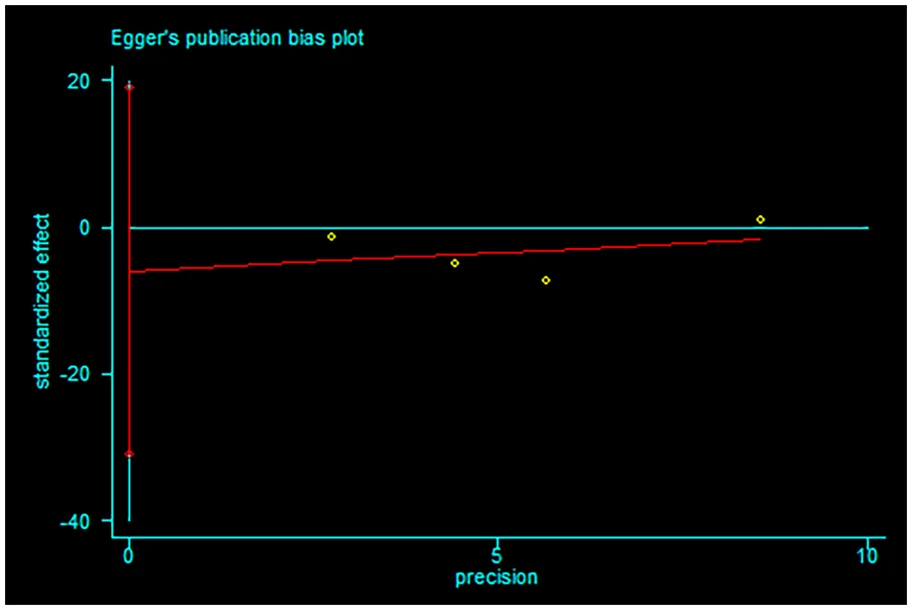
Egger's regression plot for publication bias in the analysis of continuous variables. Egger's publication bias plot. The points were symmetrically distributed around the regression line, indicating no significant publication bias (*t* = 1.08, *P* = 0.323).

#### 2.2.7 Certainty of evidence

According to the GRADE assessment (Table 3), the certainty of evidence for the categorical analysis (highest vs. lowest TMAO quartile) was low, while the certainty for the continuous analysis (SMD per 1-unit increase) was very low, downgraded for serious risk of bias and inconsistency.

**Table 3:** GRADE assessment.

Outcomes	No. of participants (studies) follow-up	Certainty of the evidence (GRADE)	Relative effect (95% CI)^a^	Anticipated absolute effects	
				Risk with low plasma TMAO	Risk difference with elevated plasma TMAO
Arteriosclerotic cardiovascular disease (ASCVD) assessed with: OR	3,820 cases, 4,972 controls (7 non-randomized studies)	⊕⊕◯◯ Low	OR 1.39 (1.21–1.61)	Low	
				0 per 1, 000	0 fewer per 1, 000 (from 0 fewer to 0 fewer)
Arteriosclerotic cardiovascular disease (ASCVD) assessed with: SMD	932 cases, 831 controls (5 non-randomized studies)	⊕◯◯◯ Very low^b^^,^^c^	SMD 0.43 (0.14– 0.71)	Low	
				0 per 1, 000	– per 1, 000 (from – to –)

CI, confidence interval; OR, odds ratio. GRADE Working Group grades of the evidence. High certainty: We are very confident that the true effect lies close to that of the estimate of the effect. Moderate certainty: We are moderately confident in the effect estimate; the true effect is likely to be close to the estimate of the effect, but there is a possibility that it is substantially different. Low certainty: Our confidence in the effect estimate is limited; the true effect may be substantially different from the estimate of the effect. Very low certainty: We have very little confidence in the effect estimate; the true effect is likely to be substantially different from the estimated effect.

a The risk in the intervention group (and its 95% confidence interval) is based on the assumed risk in the comparison group and the relative effect of the intervention (and its 95% CI).

b One included study (18) had a low NOS score (5/9). Sensitivity analysis excluding this study reduced the pooled SMD from 0.43 to 0.36, indicating that the overall estimate was influenced by the study with a high risk of bias.

c There was substantial heterogeneity across studies (*I*^2^ = 93.4%), and the direction of the effect was inconsistent [Dai et al. (16) showed a negative association, while the other four studies showed positive associations]. Even after sensitivity analysis excluding influential studies, moderate heterogeneity remained (*I*^2^ = 40.2%).

Elevated plasma TMAO compared to low plasma TMAO for ASCVD risk in adults. Patient or population: ASCVD risk in adults. Setting: Systematic review and meta-analysis of case-control studies. Cases: Patients diagnosed with ASCVD. Controls: Individuals without a documented history of ASCVD.

### 2.3 Discussion

#### 2.3.1 Interpretation of meta-analysis results

This systematic review and meta-analysis of 14 studies (including 9 in the quantitative synthesis) comprehensively evaluated the association between plasma TMAO levels and the risk of ASCVD. The pooled analysis of categorical effect sizes (highest vs. lowest quantile) demonstrated that elevated TMAO levels were significantly associated with a 39% increased odds of ASCVD (OR = 1.39; 95% CI: 1.21–1.61; *I*^2^ = 28.8%). This finding was consistent across the sensitivity analyses and showed no significant publication bias. In the continuous analysis using SMD, each unit increase in TMAO was also significantly associated with ASCVD risk (SMD = 0.43; 95% CI: 0.14–0.71); however, substantial heterogeneity (*I*^2^ = 93.4%) was observed, largely driven by two influential studies. Subgroup analyses suggested that the TMAO-ASCVD association might be more pronounced in primary prevention populations (no prior ASCVD) than in secondary prevention populations (*P* for interaction = 0.057). Notably, the certainty of evidence, as assessed by GRADE, was low for the categorical analysis and very low for the continuous analysis, primarily due to the risk of bias and inconsistency. Categorical variable analysis (highest quantile vs. lowest quantile) showed that individuals in the highest TMAO distribution had a significantly increased risk of ASCVD (OR = 1.39; 95% CI: 1.21–1.61). This finding was robust and consistent with the conclusions of most cross-sectional and prospective studies. As a key molecule produced by gut microbiota metabolism, elevated TMAO levels are closely associated with the occurrence and development of atherosclerosis (7). This finding supports TMAO as a biomarker for identifying high-risk cardiovascular status, particularly for screening high-risk populations with extremely elevated TMAO levels (7, 26–30). Importantly, the magnitude and direction of the association observed in our categorical analysis (OR = 1.39, 95% CI: 1.21–1.61 for highest vs. lowest TMAO quantile) are consistent with the seminal findings reported by Tang et al., who demonstrated a hazard ratio of 2.54 (95% CI: 1.66–3.89) for incident MACE among patients in the highest versus lowest TMAO quartile (3). While the effect size in our pooled analysis is more modest—likely due to differences in study population (primary and secondary prevention cohorts combined in our meta-analysis vs. predominantly secondary prevention in Tang's study) and outcome definitions—both studies support that elevated plasma TMAO levels are independently associated with increased ASCVD risk. Our study extends these findings by quantifying the association specifically in case–control study designs and demonstrating that the TMAO-ASCVD association is more pronounced in primary prevention populations (OR = 1.51, 95% CI: 1.28–1.78) than in secondary prevention populations (OR = 1.10, 95% CI: 0.83–1.46). Although the continuous variable analysis yielded a statistically significant pooled SMD of 0.43 (*P* < 0.001), the extremely high heterogeneity (*I*^2^ = 93.4%) indicated that the assumed linear association between TMAO levels and ASCVD risk was not consistent across studies. This lack of consistency seriously challenges the generalizability of fixed linear effects. This discrepancy may arise from differences in the baseline characteristics of different study populations (27), or the association between TMAO and ASCVD risk may be strongly modified by other factors, such as individual differences in gut microbiota composition (28). Therefore, from a clinical perspective, the results of the categorical analysis may be more applicable for identifying high-risk individuals who require targeted interventions (e.g., those with extremely elevated TMAO levels) in the general population. In contrast, the unstable and highly heterogeneous nature of the continuous analysis suggests that the incremental ASCVD risk associated with each absolute unit increase in TMAO may be relatively small in the general population or that the association is not simply linear.

Subgroup analysis further revealed heterogeneity in the association between TMAO and ASCVD risk across different prevention-level populations. The analysis showed that in the primary prevention population (i.e., those without established ASCVD), the association between elevated TMAO and ASCVD risk was significant (OR = 1.51, 95% CI: 1.28–1.78), whereas in the secondary prevention population (those with established ASCVD), the association became non-significant (OR = 1.10, 95% CI: 0.83–1.46), with a *P*-value for interaction at the margin of statistical significance (0.057). Although the continuous analysis suggested a larger point estimate in the secondary prevention subgroup (SMD = 0.54, 95% CI: −0.25–1.33), this finding was highly unreliable due to extreme heterogeneity (*I*^2^ = 96.6%) and was driven by a single low-quality study (18). After accounting for this influential study, the continuous effect in secondary prevention populations no longer exceeded that observed in the primary prevention populations. Therefore, the two sets of results are not contradictory; rather, they collectively indicate that the TMAO-ASCVD association is more robust and consistent in primary prevention settings. This important finding suggests that TMAO may play a more critical role in the early stages of atherosclerosis development. In the initial phase of the disease, TMAO participates in the initiation of atherosclerosis by influencing inflammatory responses, lipid metabolism, and other pathways (29). However, in patients with established ASCVD, its risk prediction value may be masked or attenuated by stronger traditional risk factors (e.g., low-density lipoprotein cholesterol and inflammatory markers) or the complex metabolic disturbances caused by the disease itself (30). From a clinical practice perspective, this difference has important implications: monitoring plasma TMAO levels may have greater potential value for the primary prevention of ASCVD, particularly in populations with atypical or borderline traditional risk factors (31). It may help identify high-risk individuals who are missed by traditional risk assessment models, thereby enabling early lifestyle interventions or targeted therapies, such as reducing TMAO levels by modulating the gut microbiota (32, 33). For secondary prevention populations with established disease, although the direct risk prediction value of TMAO may be limited, as an indicator of gut microbiota dysbiosis and systemic metabolic status, it may still provide clues for understanding disease progression and exploring adjunctive therapeutic targets.

#### 2.3.2 Exploration of potential sources of high heterogeneity in continuous variable analysis

Multiple studies have confirmed that plasma TMAO levels and the strength of their association with ASCVD risk differ significantly among populations. For example, a meta-analysis focusing on the Chinese and German populations showed that TMAO levels were significantly associated with the incidence and prognosis of cerebral infarction (33). This difference may originate from genetic background, dietary habits (such as the intake of red meat and eggs rich in choline and carnitine), and significant regional differences in gut microbiota composition, all of which directly influence baseline TMAO levels (7, 33). Furthermore, the interference of disease status and comorbidities cannot be ignored. The definitions and severity of ASCVD cases (e.g., stable angina pectoris vs. acute coronary syndrome) were inconsistent across studies, and renal function is a key factor affecting TMAO clearance. Renal insufficiency generally elevates TMAO levels and increases cardiovascular risk, and if studies differ in matching or adjustment for renal function, substantial heterogeneity will be introduced (34). Standardization of sample collection and processing procedures is also a source of heterogeneity. The timing of plasma sample collection (fasting or not), processing methods, storage conditions, and whether stabilizers are added may all affect the stability of TMAO measurements, leading to differences in measurement errors across studies (35).

Inconsistency in TMAO detection technology is a key technical factor contributing to the heterogeneity of the analysis. The assay methods used in different studies, such as liquid chromatography-tandem mass spectrometry or nuclear magnetic resonance spectroscopy, vary in sensitivity, specificity, and lower limits of detection, which may introduce bias in the quantification of low-concentration TMAO, thereby affecting the precision of continuous variable analysis (36). The diversity of statistical processing methods further exacerbates this heterogeneity. In the continuous variable analysis, the units of TMAO were inconsistent across studies (e.g., μmol/L and ng/mL). Some studies used raw values, while others performed logarithmic transformations to handle skewed distributions. When calculating the effect size per unit increase, some studies used a 1-standard deviation increase, while others used a 1-concentration unit increase. This inconsistency in statistical transformation is an important technical source of heterogeneity (35). Furthermore, the strategies for adjusting covariates in multivariate regression models varied greatly across studies, such as whether to adjust for renal function, dietary factors, other gut metabolites, inflammatory markers, or medication use (e.g., statins and antibiotics). Inadequate adjustment leaves residual confounding (37), while over-adjustment may remove intermediate variables in the TMAO pathway, leading to an underestimation of the effect size and thereby contributing to heterogeneity (37–40). For example, one study highlighted that multiple confounding factors, such as diet, renal function, and demographic variables, affect plasma TMAO levels, resulting in inconsistent reliability of TMAO as a biomarker across different studies (35).

#### 2.3.3 Mechanistic hypotheses explaining the difference between primary and secondary prevention

The finding that the association between TMAO and ASCVD risk differs between primary and secondary prevention populations can be explained by the stage-specific characteristics of its pathophysiological effects. In the early stages of atherosclerosis or in primary prevention populations, vascular lesions are still in the initiation or progression phase. At this stage, TMAO effectively “initiates” or “accelerates” the formation and development of atherosclerotic plaques through mechanisms such as promoting foam cell formation, enhancing platelet aggregation, and inducing endothelial inflammation (39). Therefore, during this phase, TMAO levels were relatively strongly associated with the risk of future cardiovascular events. However, in secondary prevention, patients with established ASCVD and vascular lesions often enter an advanced stage, where plaques may become unstable or even rupture, leading to clinical events such as thrombosis. At this point, the pathophysiological network is more complex and is dominated by chronic vascular inflammation, fibrosis, thrombosis, and myocardial remodeling (40–43). Although the pro-inflammatory and pro-thrombotic effects of TMAO persist, their impact may have been integrated into this broader and more intense pathological network, and the signal of its independent contribution is relatively weakened compared to other established pathological factors (e.g., complex plaques and heart failure status). This makes it statistically more difficult to detect an independent association between TMAO and subsequent event risk in patients with established diseases, resulting in a less pronounced association than that observed in primary prevention populations. This hypothesis emphasizes that the role of TMAO as a risk factor may be specific to some stages.

#### 2.3.4 Limitations of this meta-analysis

This meta-analysis had several limitations that may have affected the robustness of the conclusions. First, all included studies were observational in design, primarily case–control studies, which limited causal inference. Although a significant association was observed between plasma TMAO levels and ASCVD risk, it remains uncertain whether TMAO is a causal factor for ASCVD or merely a biomarker of disease progression (43). Residual confounding factors, such as individual differences in renal function, dietary patterns, and gut microbiota composition, may not be completely excluded, which could affect the estimation of the association (35). Second, although subgroup analyses were performed to explore the sources of heterogeneity, the extremely high *I*^2^ values in the pooled analyses indicated the presence of substantial unidentified heterogeneity. This heterogeneity may arise from racial differences in the study populations, inconsistencies in TMAO measurement methods (e.g., use of different brands of liquid chromatography-tandem mass spectrometry), and diversity in ASCVD outcome definitions (35). As mentioned earlier, significant differences exist in TMAO measurements and reporting methods across studies, such as sample processing, internal standard use, and concentration units, which constitute a major methodological limitation, restricting the comparability of the study results and the interpretability of the pooled effect sizes (35, 36). Furthermore, because of the small number of included studies, the statistical power of the publication bias tests was limited, and the possibility of publication bias could not be completely ruled out. Fifth, as with all meta-analyses of observational studies, the GRADE certainty assessment reflects the inherent limitations in the evidence base. The categorical analysis was rated as having low certainty, primarily due to the observational design of the included studies. However, the low heterogeneity (*I*^2^ = 28.8%) and consistent effect direction across studies support the robustness of the pooled estimate (OR = 1.39; 95% CI: 1.21–1.61). The continuous analysis was rated as having very low certainty, largely attributable to substantial heterogeneity (*I*^2^ = 93.4%) and the influence of a single low-quality study (Dong et al. (18), NOS = 5). Importantly, these limitations do not undermine the primary finding of the categorical analysis, which was based on a larger number of studies and showed greater stability. Our results should be interpreted as supportive evidence for an association between elevated TMAO and ASCVD risk, while acknowledging that causal inference requires confirmation in prospective cohort studies or randomized trials. Future research should prioritize the use of standardized TMAO assays and harmonized reporting units.

#### 2.3.5 Implications for clinical practice and public health

Although the evidence is not yet conclusive, the observed association between TMAO and ASCVD risk offers several potential clinical and public health implications that merit consideration. Based on the current evidence, it is not yet sufficient to recommend plasma TMAO as a routine cardiovascular risk screening marker. The reliability of TMAO as a biomarker has been inconsistent across studies, as its plasma levels are significantly influenced by multiple confounding factors, including diet, renal function, and demographic variables (35). However, for specific high-risk populations, such as patients with chronic kidney disease (CKD) or those with refractory ASCVD, measuring TMAO levels may provide additional risk stratification information to aid in clinical decision-making (44). At the public health level, emphasizing dietary interventions to modulate the gut microbiota and reduce TMAO levels represents a potential strategy for the primary and secondary prevention of cardiovascular disease. Given that red and processed meats are major dietary sources of choline and L-carnitine (2), public health efforts should target the reduction of their consumption and the intake of foods rich in choline and L-carnitine (e.g., red meat and eggs). Notably, accumulating epidemiological evidence has consistently linked high red and processed meat intakes with increased ASCVD risk. A recent large-scale meta-analysis reported that each 50 g/day increment in processed meat consumption was associated with an 18% higher risk of cardiovascular disease, and each 100 g/day increment in unprocessed red meat consumption was associated with a 9% higher risk (45). Importantly, the TMAO pathway may partially mediate this association. Studies have shown that TMAO, *γ*-butyrobetaine, and crotonobetaine together explain approximately 10% of the excess ASCVD risk associated with unprocessed red meat intake (46), highlighting a modifiable dietary target for cardiovascular prevention at the population level. However, the definitive effects of these dietary interventions on reducing the risk of hard cardiovascular endpoints (e.g., myocardial infarction and stroke) still need to be validated through large-scale, rigorously designed randomized controlled trials (47).

#### 2.3.6 Suggestions for future research directions

Future research should strive to overcome the limitations of current evidence and further explore its translational value. First, large-scale, prospective cohort studies across different races and geographical regions are urgently needed. These studies should measure TMAO levels using standardized LC-MS/MS methods and prospectively evaluate the dose–response relationship between TMAO and ASCVD events (e.g., myocardial infarction and stroke) (48–50). Study designs must adequately adjust for key confounding factors, such as renal function and baseline disease status, to accurately assess the independent predictive value of TMAO (35). Second, an in-depth exploration of the molecular mechanisms underlying the TMAO pathogenesis and individual differences is required. Research should focus on key factors in the TMAO metabolic pathway, such as the effect of genetic polymorphisms of FMO3 on individual sensitivity to TMAO and its conversion efficiency. Although the study (49) did not directly measure FMO3 activity or genotype, its findings strongly suggest that FMO3-mediated TMA-to-TMAO conversion efficiency is a key determinant of sex differences. Therefore, future research should aim to validate this mechanism by analyzing the relationship between FMO3 genetic polymorphisms, conversion efficiency, and cardiovascular events. Such precision medicine research may help achieve the individualization of cardiovascular risk prediction. Finally, interventional studies are crucial for validating the causal role of TMAO and for developing new therapies. Rigorous randomized controlled trials should be conducted in the future to assess whether reducing circulating TMAO levels through supplementation with prebiotics, probiotics, or the use of specific drugs (e.g., TMA lyase inhibitors and FMO3 inhibitors) can effectively reduce the occurrence of cardiovascular events (50). Simultaneously, exploring the potential of multi-target intervention strategies, such as traditional Chinese medicine, in modulating TMAO metabolism and improving cardiovascular outcomes represents a promising direction (44). In addition, the development of unified methodological standards is of great significance for subsequent research and data integration.

## Data Availability

The original contributions presented in this study are included in the article/Supplementary Material. Further inquiries can be directed to the corresponding author.
